# Human papillomavirus infection in patients with laryngeal carcinoma

**DOI:** 10.1186/s12885-018-4890-8

**Published:** 2018-10-20

**Authors:** Ozlem Onerci Celebi, Ebru Sener, Sefik Hosal, Mustafa Cengiz, Ibrahim Gullu, Gaye Guler Tezel

**Affiliations:** 1 Department of Otolarygology-Head and Neck Surgery, Hacettepe University School of Medicine, Ankara, Turkey; 2 Department of Pathology, Hacettepe University School of Medicine, Ankara, Turkey; 3 Department of Pathology, Ataturk University School of Medicine, Erzurum, Turkey; 4 Department of Radiation Oncology, School of Medicine, Hacettepe University, Ankara, Turkey; 5 Department of Medical Oncology, School of Medicine, Hacettepe University, Ankara, Turkey

**Keywords:** Laryngeal Cancer, Human papilloma virus, Chemotheraphy, Survival

## Abstract

**Background:**

The aim of this study was to determine the HPV positivity rate in patients with laryngeal cancer, and to determine the effect of HPV positivity on survival. An additional aim was to determine if patients with HPV positive laryngeal cancer are more sensitive to chemotherapy and if such sensitivity differs according to chemotherapy protocol.

**Methods:**

The study included laryngeal specimens obtained from 82 laryngeal cancer patients and 11 laryngeal specimens with normal laryngeal mucosa that were obtained from our hospital’s paraffin block archives between 1995 and 2013. HPV was detected via chromogenic in situ hybridization (cISH) and confirmed via genotyping.

**Results:**

HPV was not detected in any of the 82 laryngeal cancer patients’ laryngeal specimens, nor in any of the 11 archived laryngeal specimens with normal laryngeal mucosa via cISH. Genotyping confirmed these findings; none of the HPV types studied were detected in any of the specimens. As none of the study samples were HPV positive, it was not possible to compare survival, recurrence, or chemotherapy sensitivity.

**Conclusions:**

HPV infection is not a leading cause of laryngeal cancer; however, additional research on HPV positivity in patients with laryngeal cancer and its effect on recurrence, survival, and chemotherapy sensitivity is warranted.

## Background

Laryngeal cancer is the 20th most common cancer in Europe, with around 39,900 new cases diagnosed in 2012 (1% of the total) [1]. Laryngeal cancer is known to be caused primarily by tobacco and alcohol consumption; however, viral infections including human papillomavirus (HPV) have also been associated with head and neck cancer [2–6]. Laryngeal cancer differs from the other head and neck cancers in that several studies on HPV positive and HPV negative laryngeal cancer patients reported no difference in chemotherapy sensitivity and survival [7–9].

Although the reported incidence of HPV positivity in laryngeal cancer patients varies [9–12] fewer data are available for laryngeal cancer, as compared to oropharyngeal cancer, in terms of HPV infection, survival, and chemotherapy sensitivity. Moreover, there is a lack of data on the frequency of HPV positivity in patients with laryngeal cancer in Turkey. The aim of the present study was to determine the HPV positivity rate in patients with laryngeal cancer, and to determine the effect of HPV positivity on survival. An additional aim was to determine if patients with HPV positive laryngeal cancer are more sensitive to chemotherapy and if such sensitivity differs according to chemotherapy protocol.

## Methods

### Patient demographics

Retrospective data analysis was performed to include patients with laryngeal cancer between 1995 and 2013. The patients who had laryngeal squamous cell carcinoma, who had a biopsy spesimen available in the Pathology Paraffin Block Archives, who received chemoradiotherapy as the first line treatment were included. All patients underwent chemoradiotherapy post diagnosis, and all chemotherapy protocols included an alkylating agent. Patients that received any treatment before direct laryngoscopy and biopsy, had surgery as the primary treatment procedure, received only radiotherapy, and those with follow-up < 2 years were excluded. 82 patients met the criteria and the formalin-fixed paraffin-embedded tissue specimens (FFPE) and slides of tumors stained with H&E for each patient were retrieved. All patient slides were re-examined for confirmation of the diagnosis.

The retrospective data analysis included demographic and clinical findings, as well as the histopathological characteristics of the tumor. Grade of differentiation, primary tumor extension, and nodal status, were recorded. Treatment regimens and the recurrence status were also assessed. Disease-free survival (DFS) was defined as the time from the date chemoradiotherapy was initiated to the time recurrence was detected.

The control group comprised of 11 laryngeal specimens reported as normal laryngeal mucosa obtained from the Department of Pathology Paraffin Block Archives. These pathological specimens included 6 male and 5 female patients. The mean age of these subjects was 47.45 years, ranging from 35 to 62 years. These 11 patients had undergone endoscopic laryngeal surgery for benign lesions. These lesions were resected and biopsied during the direct laryngoscopy procedure. These biopsy specimens were reported to be normal laryngeal mucosa without evidence of a premalignant or a malignant lesion.

HPV was detected via chromogenic in situ hybridization (cISH) and was confirmed via genotyping. The study was approved by the University Ethics Committee and was conducted in accordance with the Declaration of Helsinki.

### HPV DNA detection via in situ hybridization (ISH), HPV DNA detection and typing

High-risk (HR)-HPV DNA in-situ hybridization (ISH) was done using proprietary reagents (Inform HPV III Family 16 Probe [B], Ventana Medical Systems, Inc., USA), which can detect high risk HPV genotypes (HPV 16, 18, 31, 33, 35, 39, 45, 51, 52, 56, 58 and 66) [13]. HR-HPV(+) cervical specimens were used as the positive control group and HR-HPV(−) cervical specimens were used as the negative control group. The HR-HPV ISH test was scored as positive if there was any blue reaction product that co-localized with cell nuclei [13]. The staining was reported as positive if it showed diffuse nuclear and cytoplasmic staining or punctate nuclear staining. Staining that was pale and limited to the nucleoli of cells were reported as negative.

Flow-through hybridization was performed for HPV genotyping, using an HBGA-21 GenoArray Diagnostic Kit (HybriBio Biotechnology Ltd. Corp., Chaozhou, Guangdong Province, China) in accordance to the manufacturer’s instructions. The HPV GenoArray helps the identification of 21 HPV genotypes (HPV 6, 11, 16, 18, 31, 33, 35, 39, 42, 43, 44, 45, 51, 52, 53, 56, 58, 59, 66 and 68) and CP8304. Of the 21 genotypes, 5 were classified as low risk (HPV 6, 11, 42, 43, 44) and the remaining HPV genotypes are classified as high-risk [14].

### Statistical analysis

HPV positivity and all clinicopathological data, including age, gender, tumor stage, differentiation, tumor localization, and tobacco and alcohol use, underwent descriptive statistical analysis using SPSS v.10.0 for Windows (SPSS, Inc., Chicago, IL).

## Results

### Patient demographics and clinicopathological findings

The study included laryngeal specimens obtained from 82 patients with laryngeal cancer and 11 laryngeal specimens reported as normal laryngeal mucosa. Among the 82 laryngeal cancer patients, 78 were male and 4 were female; mean age was 56.6 years (range: 40–75 years). Tumor localization included the supraglottis (*n* = 58 [70.7%]) and glottis (*n* = 24 [29.3%]). In all, 5 (6.1%) patients were clinically stage II, 38 (46.3%) were stage III, and 39 (47.6%) were stage IV. In total, 46 (56.1%) patients had no evidence of nodal involvement at the time of diagnosis. Tumors were histopathologically classified as well differentiated carcinoma (*n* = 33 [40.2%]), moderately differentiated carcinoma (*n* = 44 [53.7%]), and poorly differentiated carcinoma (*n* = 5 [6.1%]). Table 1 summarizes the clinical and pathological findings of the patients. A combination of radiotherapy and chemotherapy was administered to all patients. Of all patients, 46 received induction chemotherapy followed by concomitant chemoradiotherapy whereas 36 received only concomitant chemoradiotherapy. All chemotherapy protocols included an alkylating chemotherapeutic. The chemoradiotherapy protocol is shown in Table 2. During follow up, 35 (42.7%) patients had recurrence; the mean time from diagnosis to recurrence was 20.6 months (range: 6–50 months). Mean duration of follow-up in the patients without recurrence was 55 months (range: 24–120 months). Patients who had less than 2 year follow up were excluded. However, 1 of the patients who died 15 months after treatment with no evidence of recurrent disease and was included among the patients without recurrence. All 82 laryngeal cancer patients had a history of tobacco use, but most did not remember how long they smoked or how much they smoked; therefore, data were not analyzed according to quantity and duration of smoking.

**Table 1:** Clinical and pathological characteristics of the patients

Characteristics^a^	Value
Age (years)	
Mean	56.6
Range	40-75
Gender	
Male	78 (95.1)
Female	4 (4.9)
Primary Tumor Site, No. (%)	
Supraglottis	58 (70.7)
Glottis	24 (29.3)
T Stage, No. (%)	
T2	14 (17)
T3	53 (64.7)
T4	15 (18.3)
N Stage, No. (%)	
N0	46 (56)
N1	10 (12.2)
N2a	3 (3.6)
N2b	10 (12.2)
N2c	10 (12.2)
N3	3 (3.6)
Stage, No. (%)	
Stage II	5 (6.1)
Stage III	38 (46)
Stage Iva	35 (43)
Stage IVb	4 (4.9)
Histopathological Differentiation, No. (%)	
Well-Differentiated	33 (40.2)
Moderately-Differentiated	44 (53.7)
Poorly-Differentiated	5 (6.1)
Chemotherapy Regimen, No. (%)	
Induction CT + concomitant CRT	46 (56.1)
Concomitant CRT	36 (43.9)
Recurrence, No. (%)	
Recurrence (+)	35 (42.7)
Recurrence (–)	47 (57.3)

^a^Data are expressed as No (%) unless otherwise indicated

**Table 2:** Chemotherapy protocols according to patient groups

	Chemotherapeutic Agent	Dose	Days
Group 1 (Induction CR + CRT)			
Induction Chemotherapy^a^	*Cisplatin*	75 mg/m^2^	1st
	*Docetaxel*	75 mg/m^2^	1st
	*5-FU*	750 mg/m^2^	1st-5th
Concomitant Chemotherapy^b^	*Cisplatin*	75 mg/m^2^	Once in 7 days
Group 2 (CRT)			
Concomitant Chemotherapy^b^	*Cisplatin*	75 mg/m^2^	Once in 7 days

^a^Patients received this protocol once in 3 weeks for 3 times. Some patients received the combination of cisplatin and docetaxel and others received the combination of cisplatin and 5-FU. Doses were modified in the range of 20–25%, based on general health status and organ functions of the patients

^b^Patients received this protocol during radiotherapy treatment

*CT* Chemotherapy, *CRT* Chemoradiotherapy

### In-situ hybridization and genotyping

Chromogenic In-Situ Hybridization (cISH) was performed to assess HR-HPV positivity. cISH findings were evaluated by 2 independent observers. None of the 82 laryngeal specimens obtained from patients with squamous cell carcinoma and none of the 11 laryngeal specimens with normal laryngeal mucosa exhibited a positive cISH staining pattern. Genotyping confirmed the cISH findings; none of the HPV types studied were present in any of the specimens.

## Discussion

HPV infection has been shown to cause oropharyngeal cancer. However, the clinical significance of HPV infection in head and neck squamous cell cancer other than oropharygeal cancer is yet to be determined. The prevalence of laryngeal and hypopharyngeal squamous cell carcinomas that are HPV positive varies from 0% [2, 15, 16] to 75% [17]. This difference in the prevalence of HPV in laryngeal cancer has been explained by the sensitivity of diagnostic techniques, ethnic and geographical differences in patients, small study samples, low quality of the specimens, and differences in methods of sample storage and lesion localization [7, 11].

PCR and ISH are widely used HPV detection systems. ISH is commonly used to detect HPV in clinical biopsy specimens. Some studies report that PCR-based methods are more sensitive than ISH. Besides, PCR-based methods can yield false positive results, because they may not differentiate biologically irrelevant HPV from clinically significant HPV, whereas ISH correlates with biologically and transcriptionally active HPV, differentiating between clinically significant and non-tumorigenic HPV DNA [18–21]. HPV DNA PCR amplification only shows the existence, whereas ISH can show the integration of the viral DNA into the host genome [12, 22].

In the current study we used HR-HPV chromogenic ISH for detection of HR-HPV; however, none of the 82 laryngeal specimens of squamous cell carcinoma and none of 11 laryngeal specimens of normal laryngeal mucosa exhibited a positive cISH staining pattern. Although the reported prevalence of HPV positive squamous cell cancer of the larynx and hypopharynx varies widely, the literature generally indicates that the prevalence is higher than that observed in the present study, but some studies have also reported no HPV positivity or a very low prevalence of HPV positivity [2, 15, 23–25]. The study by Castellsague et al. including 1042 laryngeal cancer patients from 29 countries tested the specimens with PCR and a DEIA for the presence of HPV-DNA and samples containing HPV-DNA were further subject to HPV E6*I mRNA detection and to p16INK4a, pRb, p53, and Cyclin D1 immunohistochemistry [25]. This study, the largest exploring HPV attribution in head and neck cancers also found a small percentage of HPV positive laryngeal cancer cases. Some studies report that PCR-based methods are more sensitive than ISH and that PCR-based methods can yield false positive results [15, 18–21]. Such false positivity was avoided in the present study via use of cISH, which could be one of the reasons why HPV positivity was not observed in any of the present study’s laryngeal specimens. Gallo et al. [15] examined the role of HPV virus in laryngeal cancer using PCR and taking all necessary precautions to avoid false positive, as well as false negative findings. They reported that none of the 40 cases of squamous cell carcinoma showed presence of HPV genome, which supports the notion that earlier reports of high prevalence of HPV positivity might have been due to false positive results, and that many the majority of the laryngeal cancers are not related to HPV infection, as the current findings indicate.

The effect of tobacco use on laryngeal cancer is well-known, and we also reviewed the data and/or asked the patients about their tobacco use. Although the data were not very reliable because they were collected via self-report and the patients did not remember precisely how long they smoked or how much they smoked, all our patients had a history of tobacco use. Gheit et al. [23] reported that 75% of their patients harboring viral DNA had a history of tobacco use, and suggested that tobacco use could act together with HPV induced cancer formation. In the present study all patients had a history of tobacco use, but no HPV DNA was detected in any of the laryngeal specimens.

Some studies regarding head and neck cancer found out that HPV positivity was observed in younger patients compared to patients with HPV negative cancer [3, 4]. The current study’s patients were aged 40–75 years; none were considered young. Based on those earlier reports, it is possible that had the present study included younger patients, some with HPV positivity would have been identified [3, 4]. The transmission of HPV has been widely studied, and orogenital sexual contact and multiple sex partners were shown to increase the rate of transmission of the virus [25]. Roshan et al. [2] conducted a study in Iran and reported that HPV was not encountered in any of the specimens; neither in biopsies of patients with laryngeal cancer nor in biopsies obtained from healthy people, as in the present study. They attributed their findings to the rarity of high-risk sexual behavior in Iran. Their study differs from the present study as they only investigated HPV 16 and 18, whereas the present study investigated all HR-HPV types; in addition, the present study’s patient group was larger.

Earlier studies on HPV positivity in laryngeal cancer in Turkey reported that 7.4% [26] and 10.6% [24] of patients had HPV DNA. The difference in those reported percentages and that found in the current study might be secondary to differences is diagnostic techniques; Guvenc et al. [24] used hybrid capture to detect HPV and Gungor et al. [26] used PCR genotyping. These studies also included LR-HPV infections—another possible cause for the differences in findings, as when only HR-HPV infection was considered, the HPV positivity rate in Gungor et al.’s study was only 1% [26]. Other studies reported low rates of HPV positivity in normal laryngeal mucosa samples, of which many cases showed presence of low-risk HPV [12, 15, 18]. Similarly, none of the present study’s normal laryngeal mucosa specimens were HPV positive.

The present study is among the few from Turkey to investigate HPV infection in patients with laryngeal cancer, and compare it to HPV positivity in normal laryngeal tissue. The cISH technique used in the present study is not widely used for studying laryngeal cancer. A strength of the present study is that HPV negativity based on cISH was confirmed via genotyping. As some earlier studies reported, HPV positivity was not observed in any of the present study’s laryngeal specimens (both cancerous and normal) [2, 15, 16] indicating that HPV does not play a major role in the etiology of malignant laryngeal lesions. Nonetheless, the present study included only patients over 40 years old and a small overall population, which might be considered limitations. Lastly, as none of the present study’s laryngeal specimens were HPV positive, it was not possible to obtain any data on the effect of HPV positivity on recurrence, survival, or chemotherapy sensitivity.

## Conclusion

The present findings suggest that HPV infection does not play a major role in laryngeal cancer; however, additional research is required to increase our understanding of the prevalence of HPV positivity in laryngeal cancer patients and the effect of HPV positivity on recurrence, survival, and chemotherapy sensitivity.

## Abbreviations

cISH: Chromogenic In situ hybridization
DEIA: DNA enzyme immunoassay
DFS: Disease Free Survival
FFPE: Formalin-fixed paraffin embedded
HPV: Human Papilloma Virus
HR-HPV: High-risk Human Papilloma Virus
IHC: Immunohistochemistry
LR-HPV: Low-risk Human Papilloma Virus
PCR: Polymerase Chain Reaction
PPY: pack per year
SCC: squamous cell carcinoma
